# Policymakers’ Research Capacities, Engagement, and Use of Research in Public Health Policymaking

**DOI:** 10.3390/ijerph182111014

**Published:** 2021-10-20

**Authors:** Natasa Loncarevic, Pernille Tanggaard Andersen, Anja Leppin, Maja Bertram

**Affiliations:** Unit for Health Promotion Research, Department of Public Health, University of Southern Denmark, Degnevej 14, 6700 Esbjerg, Denmark; ptandersen@health.sdu.dk (P.T.A.); aleppin@health.sdu.dk (A.L.); mbertram@health.sdu.dk (M.B.)

**Keywords:** evidence-informed policy making, public health policy, SPIRIT Action Framework, capacity-building, research engagement action, research use, Denmark

## Abstract

The use of research in public health policymaking is one of the prerequisites for successfully implemented health policies which have better population health as an outcome. This policy process is influenced by the actors involved under the policy umbrella, with inter-related contextual factors and specific structural and institutional circumstances. Our study investigates how policymakers’ research capacities influence the use of research in the health policy process and identify areas where capacity-building interventions give the most meaning and impact. Furthermore, we investigate policymakers’ research engagement and use this to inform public health policy in the public sector in Denmark. We collect and report data using Seeking, Engaging with, and Evaluation Research (SEER) methodology. Policymakers are reported to have research capacity, but it is questionable how those competences have actually been used in policymaking. Decision-makers were often not aware or did not know about the existing organizational tools and systems for research engagement and use and two third of respondents had not been part of any research activities or had any collaboration with researchers. Overall, research use in public health policymaking and evaluation was limited. As a conclusion, we propose that capacity-building interventions for increasing research use and collaboration in EIPM should be context-oriented, measurable, and sustainable in developing individual and organizational competences.

## 1. Introduction

**Figure 1:**
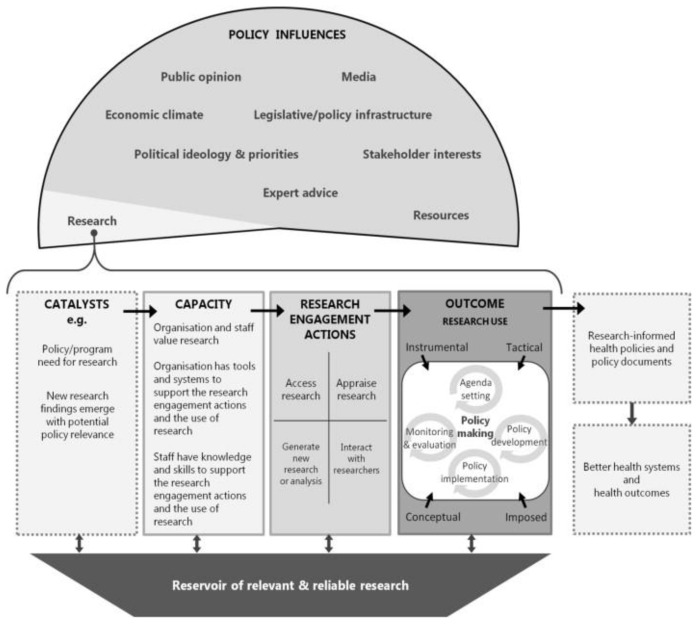
The SPIRIT Action Framework (Reprinted from The SPIRIT Action Framework: A structured approach to selecting and testing strategies to increase the use of research in policy. Soc Sci Med, (2015), 136–137, 147–155. Redman, S., Turner, T., Davies, H., Williamson, A., Haynes, A., Brennan, S., Green, S. Copyright (2021), with permission of Elsevier).

Traditionally, policymaking is defined as a process of prioritizing, planning, implementing, and evaluating policy initiatives [1,2,3,4]. As in all policy processes, the health policy process is influenced by the actors involved, inter-related contextual factors, as well as structural and institutional circumstances [3]. Research evidence is therefore only one factor contributing to policy decisions [5,6,7,8]. All these different kinds of influences are well presented by the SPIRIT Action Framework [9] (see Figure 1). This framework is based on and covers recent theories on research engagement and use, knowledge exchange and the connections to policymakers’ research capacities.

Health policymaking should be well-informed and supported by the best available research evidence [6,10] and policymakers’ expertise. At the same time, community characteristics, needs, and preferences should be considered [11]. This approach, known as Evidence-Informed Policymaking (EIPM), is rooted in evidence-based public health [3,6,10,12,13,14,15]. It follows the primary principles of health promotion and accommodates complex health policy processes [16,17]. EIPM acknowledges that evidence for this policy originates from research but also includes knowledge from other sources such as needs assessments, population characteristics, community resources and values, ideas and interests, professional and practical experience, all in a broader environmental and organizational context [3,6,12,13,14,17,18,19,20,21,22,23]. The aim of using the EIPM approach is to improve health systems’ performance and the health of the population by developing more effective and efficient public health policies [1,2,3,24,25,26,27]. In this process, stakeholders should be able to use research evidence more easily and tailor health promotion interventions to the needs of their communities. However, it is still unclear when the EIPM process is ‘well or enough evidence-informed’ to reach its aim [28,29,30]. In the present article our interest is to further investigate EIPM and, in particular, the use of research evidence by health policymakers in this process.

The Knowledge-to Action Cycle Framework suggests that research may be used at any stage of a policymaking process [14,31]. Furthermore, Bowen and Zwi’s Framework for Action illustrates the pathway to EIPM through the active sourcing, use and implementation of the evidence [6]. They suggest using evidence in all phases of policy progression, from idea to implementation. Meanwhile, Haynes and colleagues define research as one critical source of evidence among many, with the potential to guide the health policy process and hold governments accountable when (if) they make mistake [32]. Moreover, combining research evidence with other types of evidence is not simply about summarizing results and communicating them to inform policymaking; it is the co-creation and exchange of knowledge between society and the scientific community for mutual benefits [33].

The co-creation and exchange of knowledge in EIPM typically happens through the collaboration and interaction between researchers and policymakers, who often have different world views, capacities, abilities, willingness, and time frames within their work [25,34,35]. Researchers and policymakers are two disparate parties acting as “travelers in a parallel universe” or “coming from different planets-researchers from Mars and policymakers from Venus” [33,36,37], where researchers supply the policymakers with on-demand research [38]. However, researchers’ efforts to produce new knowledge and communicate it to policymakers are not sufficient if the policymakers do not find it relevant, timely, accessible, or communicated in a way which is primed for policy implementation [34,36,39,40]. Furthermore, if the policymakers are not receptive to research (e.g., have no confidence in using research, no access to research, no capacity or competencies to apply research, or are missing the organizational value of research), it is unlikely that even the best available research evidence will be able to inform policies [41,42].

The literature suggests that individual policymakers are critical participants in decisions regarding the use of evidence, and that these decisions are influenced by personal qualities and capacities [11,41,43]. Therefore, one of the most crucial enabling factors in using research evidence in public health policymaking might be research capacity. In the present study, (research) capacity is defined according to Newman et al. (2012) and Brennan et al. (2017): “Capacity is a widely used term conceived as a multi-level concept (individual, organizational, enabling environment) encompassing four elements: tools, skills, staff, and infrastructure (and roles in it), increasingly used to build competencies to implement evidence-based practice.” Campbell and Moore’s review on using research in health policy drew the conclusion that building individual capacities may help policymakers understand and use research in their policy decisions [44].

However, it is not only the policymakers’ research capacities that influence the integration of research evidence into health policies. Aside from the knowledge exchange and co-creation with researchers, organizational system support for research engagement actions in health policymaking (e.g., tools and systems) also plays a relevant role and has a significant influence in EIPM [9,11]. Hence, it is important to consider the specific contexts in which policymakers develop and implement health policies [6,7,45].

**Figure 2:**
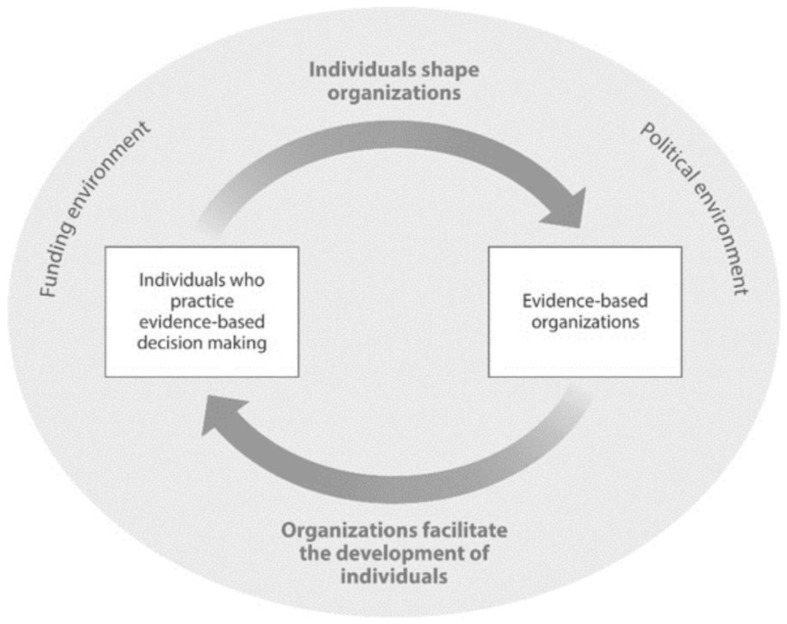
Relationships between people and organizations in public health decision making (Reprinted from The Relationships Between State Health Department Practitioners’ Perceptions of Organizational Supports and Evidence-Based Decision-Making Skills. Public Health Reports (1974), Mazzucca, S. et al. (2021), Copyright (2021), with permission from SAGE Publications, Inc.).

Some researchers define a positive research culture as a supportive environment within the organization that enables and supports research to generate new knowledge and translate research into practice [46,47]. This is critical in EIPM and essential for building an individual and organizational research capacity. In this article, we use the SPIRIT Action Framework (see Figure 1) to assess policymakers’ research capacities and the engagement in and use of research. Another framework that has been applied is Mazzucca et al.’s framework that states: “As people shape organizations and organizations support individual skill development, overall capacity for evidence-based public health can improve.” [11] (see Figure 2).

Even though research capacity has been targeted by a few studies in the capacity building area, it is still a neglected area of policy analysis and research efforts to date (e.g., hard to measure and lacking agreement on definition) [6,7,46]. There is a lack of empirical findings, especially in Europe, on policymakers’ research capacities and motivations regarding research evidence. The largest body of published evidence is from Australia, as well as from different developing countries, particularly from Africa [7,9,46,48,49,50,51,52,53].

This study aims to investigate the association between research capacity and research use among health policymakers in Denmark so that areas for improvement can be identified and targeted capacity-enhancing interventions can be developed.

In the following text, the term “health policymakers” refers to public health policymakers, and the public health “policymaking process” includes drafting, writing, developing, contributing to health policy, programs, or strategy.

## 2. Method

### 2.1. Study Design

This article’s results are part of a cross-sectional survey study conducted in 2018 and 2019 in Denmark among health policymakers.

### 2.2. Setting and Participants

Eligible participants in the survey were adults (18+ years) health policymakers, managers (e.g., hospital and municipality-based managers), and decision makers from non-governmental organizations who were part of policymaking process/es. We recruited participants from membership lists of the Danish Society of Public Health and the Danish Healthy Cities Network. To our knowledge, these are the organizations with the highest number of health policymakers in Denmark.

The Danish Society of Public Health is an organization with nearly 800 members who have different professional profiles (not all are policymakers). They share the same aim, “to promote public health, prevent diseases and reduce the impact of diseases as well as to reduce health inequalities between different groups of the Danish society”. The Danish Healthy Cities Network is a “network of municipalities and regions politically committed to cooperation to strengthen and develop the field of public health locally,” and 56 of 98 Danish municipalities are members.

An exclusion criterion was being a researcher working within a university or other research organization at the time of data collection.

All potential participants received an e-mail invitation containing an explanation of the survey and a link to the self-administered questionnaire. There were two rounds of data collection; the first was from 19 December 2018 to 31 January 2019, and the second was from 27 September 2019 to 18 October 2019. The second round was necessary to increase the number of responses.

To adhere to data protection rules, secretaries of the Danish Society for Public Health and the Danish Healthy Cities Network forwarded the e-mails to everyone on their membership lists. During both data collection rounds, reminder e-mails were sent two and three weeks after the initial invitation e-mail.

Survey respondents could access the questionnaire after reading basic information about the survey (e.g., survey goals, confidentiality, and use of collected data) and after ticking a box indicating informed consent.

Respondents with policymaking experience in the last 12 months had the opportunity to answer all questions. Respondents who had not been part of a policymaking process 12 months prior to the survey were not eligible to answer the questions from domains “Research Engagement Actions” and “Research Use” in policymaking. The questionnaire contained a skip function that skipped the participant to the “without policymaking experience in the last 12 months prior the survey” questions.

Participation was voluntary and anonymous, with no personal or other sensitive data included.

### 2.3. Survey Instrument

The survey measured the individual capacity to engage with and use research in policy development. The questionnaire was based on the SEER-instrument (Seeking, Engaging with, and Evaluation Research: [7,9,54]. The SEER assessment measure is one of three instruments developed under the SPIRIT Action Framework to help targeted capacity-building interventions. SEER measures “*the perceptions of individual policymakers focusing on the value they place on using research, their confidence in their knowledge and skills to use research, and the extent to which their organization supports the use of research. Measuring these factors provides data needed to identify priority areas for intervention and to evaluate the effects of capacity building interventions.*” [7]. The SEER tool has shown acceptable reliability and validity in Australian surveys but has so far not been used in a European context.

Before SEER translation and adjustments for the Danish context, we obtained approval to use this tool from the Australian authors.

Survey translation and adjustments occurred in several steps. First, Danish public health professionals with proficient English knowledge translated the survey into Danish. An independent researcher with knowledge of both English and Danish translated the survey back to English to determine the translation’s effectiveness. Discrepancies were solved in subsequent joint consensus meetings. The last step of this translation process was a pre-test of the newly translated and adjusted survey in Danish.

The first pre-test was conducted with policymakers from Danish municipalities, hospital managers, NGO members, and researchers from the university (six participants in total). This test confirmed that the survey was easy to understand, words and phrases were meaningful, and the scientific level was appropriate. Further, it was ascertained whether the questions covered the relevant topics to a sufficient degree or whether more questions were required. Participants wrote edits and comments into the text and these comments were discussed in phone consultations and used to adapt the questionnaire.

A second questionnaire pre-test with three members of the study population determined distribution readiness. This version also contained background information and the consent form.

### 2.4. Measurements

The SEER questionnaire consisted of 61 questions/items covering four domains, answered partly on Likert scales, partly on binary response scales, and partly with open response formats (e.g., name of the health policy which the policymaker was involved in 12 months prior survey).

The four main domains encompassed: general demographic variables, policymaker capacity to use research, research engagement actions, and actual research use in the health policymaking process. Three principal domains contained further subdomains. Thus, the domain ‘Policymakers’ capacity to use research’ contained four subdomains covered by 26 questions/items. The ‘Research engagement actions (REA)’ domain contained five subdomains with 18 questions, and the ‘Research use (RU)’ domain contained two subdomains with eight questions (see Supplementary Material File S1: Annex I and File S2: Annex II).

Mean ratings above 3.5 on the 1–7-response scale were considered as sufficient or desirable action, while scores below 3.5 were deemed limited, indicating potential deficits and the need for contextually tailored capacity development [50].

For analysis purpose, individual items of the three principal domains were combined into new variables (see Supplementary Material File S1: Annex I and File S2: Annex II), i.e., scores on the individual items were totaled and subsequently divided by the number of items to arrive at a mean score. Thus, for the research capacity domain, 26 questions/items with continuous scales were summed up into four new variables for four subdomains (Individual value placed on using research; Individual confidence in own skills and knowledge; Organization value placed on using research; Organizational tools and systems to support REA and use). Similarly, the six items of REA’s domain “interaction with researchers in the last 12 months”, which consisted of six questions, were summed up to one variable. In the same vein, the four individual items for the RU domain, “extent of research use”, were summed up to one variable. Cronbach’ alphas for the respective subscales were in the range from 0.76–0.91.

### 2.5. Data Analysis

Data analysis was conducted with the help of IBM SPSS 26 for Windows [55].

For the rating scale responses, the two highest (strongly agree and disagree), as well as the two lowest response options (strongly disagree and disagree), were collapsed because only very few respondents had chosen the highest or lowest response option. Therefore, the original seven-point scales were reduced to five-point formats. For those items that contained a ‘don’t know-response option’, these ‘don’t know’ answers were combined with ‘never’ or ‘no’ responses and summarized as “no”, because it was assumed that, for instance, not knowing the value that one’s organization placed on the use of research would have a similar (lack of) effect on the use of research as being aware that no value is placed on using research. Additionally, for some demographic variables, response categories were collapsed, e.g., age range, level of education, years of working (see Supplementary Material File S3: Annex III).

Descriptive statistics were used to analyze the data and results are presented as means, median, and standard deviations for continuous scales and as percentages for dichotomous variables.

## 3. Results

### 3.1. Participants

We sent the questionnaire to 715 members of the Danish Society for Public Health and the Healthy Cities Network. Five members refused participation by not giving informed consent, 586 did not respond, 111 did not complete the survey, and 113 finished it. Twenty-two survey respondents had to be excluded since they reported that they were working for research organizations. The final tally of respondents for analysis was *n* = 91. Due to the General Data Protection Regulation (GDPR) procedures, we could not access the member lists of Danish health policymakers from the two organizations the participants were recruited from. Therefore, it was not possible to ascertain the actual number of policymakers among the members to compute a response rate.

### 3.2. Sample Characteristics

Table 1 shows the general demographic characteristics of the respondents. Most participants were female (68%), two-thirds of the respondents were over 40 years old, half of the respondents (51%) had a short higher education, and almost half (44%) worked in a municipality. The other 38% were regional administrative policymakers, and 18% reported working in non-governmental organizations, consultancy groups, and private companies. Almost one-third had worked in their current organization for more than 11 years. Two-thirds of all responders (62%) had worked on writing, drafting, and developing health policies, strategies, and documents in the last 12 months.

### 3.3. Research Capacity (RC)

Table 2 shows the means, standard deviations, and medians for the policymakers’ answers about their research capacities.

The highest ratings were related to the two subdomains “Value that individual policymaker places on using research” and “Confidence that individual policymaker has in knowledge and skills to use research” (M = 4.0 and M = 3.9). Judgements of the “Value that the organization places on research use” were lower, with a mean of 3.41 indicating a potential need for improvement. Very low ratings became apparent for the subdomain “Tools and systems which the organization has available to support research engagement actions” (M = 2.11). Almost two-thirds of respondents did not know or answered ‘no’ to the questions of whether their organization provided guidance on how research should be used, had systems in place that encouraged the organizational leaders to support the use of research, provided access to training in the use of research and methods to review the research, or documented processes for how the health policies should be evaluated. Nevertheless, 72% stated that they were aware that their organization “has existing relationships or methods for engaging/collaborating with research organizations” (see Supplementary Material File S4: Annex IV).

### 3.4. Research Engagement Actions (REA)

Table 3 presents the frequencies and percentage results for specific research-related activities and interactions with researchers reported by 56 responders (62% of the sample) who had worked in writing, drafting, and developing health policies, strategies, or programs in the 12 months preceding the survey. For the health policy they had worked on, 61% of the respondents had searched for published “reviews of research” (e.g., systematic reviews, meta-analysis), while only 23% reported that they had themselves conducted a review. Fifty-seven percent stated that they had searched for primary study results (e.g., RTC, qualitative studies) that could have helped them in the policymaking process which they had been involved in.

As for the specific aspects of assessment of methodological quality, the majority of participants indicated that they had used criteria such as the appropriateness of the method used (65%), unbiased/reliable results (71%), and the generalizability of the findings in context (94%).

Around two-thirds of the respondents had not been involved in any kind of research activity, either alone or in collaboration with researchers, while being part of the policymaking process. The data in Table 2 and Supplementary Material File S4: Annex IV revealed that they had either never interacted or interacted only once with researchers about the policy or research direction, collaboration or research grant implementation, publication contribution and/or about being a part of a research team in the preceding 12 months (M = 1.96).

### 3.5. Research Use (RU)

Table 2 shows the answers regarding the extend of research use in policymaking phases by 56 responders (who had worked in writing, drafting, and developing health policies, strategies, or programs in the 12 months preceding the survey) (M = 3.09).

Table 3 shows the answers for the four types of research use (conceptual, instrumental, tactical, and imposed). More than half (57%) of the respondents did not use the research to understand and reflect on the health policy topic of interest. Conversely, most (82% and 86%) used it to support decision making about health policy content or direction and to convince others about health policy action. Twenty-seven percent reported “imposed research use”, that is they said that their organisations explicitly required them to use research in health policy development.

## 4. Discussion

The survey results provided a critical insight into Danish health policymakers’ research capacities, individual levels of research engagement actions, and use of different types of research in health policymaking [9]. We discuss each of these three principal domains separately.

### 4.1. Research Capacity

We measured research capacity through four subdomains (see Figure 1, Supplementary Material File S1: Annex I, File S2: Annex II): (1) the value that the individual policymaker places on using research, (2) the confidence individual policymakers have in their knowledge and skills for research engagement actions and use, (3) the value the organization places on research, and 4) the tools and systems the organization has to support research engagement actions and use. The level of Danish policymakers’ research capacities was comparable to that reported by other international studies [52]. However, this contrasted with previous Danish research, which signaled *a lack* of perceived individual capacity, knowledge, and research skills for health policymaking [4,56,57,58], whereas the participants of the present study expressed confidence in their own knowledge and skills to use it.

We can argue that previous small-scale, capacity-building interventions among Danish policymakers might have brought about this change [18]. Nevertheless, it is essential to be sustainable in competencies, motivation, and have a positive attitude about improving research capacity and use in future policymaking [7,36,39].

Furthermore, the results are in line with Australian data [52] in that they identified two areas for potential improvement in relation to research capacity.

The first area is how policymakers’ organizations value the use of research evidence in policymaking. Furthermore, our results here are consistent with prior published data pointing out that a strong belief of organizational leaders in the importance of using research for health policymaking and their expectation of its usefulness for evaluating policies is promoting research use [4,18]. Moreover, Danish policymakers reported that they are not encouraged to interact or collaborate with researchers or research organizations in their concrete policymaking process. Innvaer et al. (2002), and Lavis et al. (2005) suggest that interactions between researchers and policymakers build trust and increase the chance of policymakers using research [36,39]. Green and Bennett, as part of the WHO efforts, stated that sufficient contact and interaction between researchers and policymakers is critical in order to bridge gaps between them [59].

Second, a potential area for capacity intervention has been identified in relation to the subdomain:” tools and systems that (policymakers’) organization provides to support research engagement actions and use.”. The ratings here were very low, consistent with prior literature [18,60]. Two-thirds of all respondents did not know if their organization had standard guidance and support tools to use research in policymaking and methods for conducting research reviews and documenting policy evaluation processes. This indicates that those working in policymaking organizations are in a difficult position. They are aware of the fact that their organizations believe in implementing and using research in their work and health policies, but at the same time these organizations do not provide the tools and support systems to do so. An Australian study, which used the same methodology as the present study to assess baselines before capacity-building interventions, showed a significant increase in the confidence in using research when the support by organizational tools and systems was increased [52].

Campbell and Moore, in a rapid review, found that just a few studies investigated the importance of organizational managers supporting research use in policymaking [44]. Moreover, this review stated that the improvements needed for the capacity-building interventions were not sustainable after a six-month follow-up. Hawkes and colleagues showed that building individual and organizational research capacity (to access, understand and use research was useful and successful, and turned out to be a prerequisite for long-term change at the system level [61]. Capacity building interventions on an individual level were more acceptable, methodologically more feasible, and more easily measured than on the other two levels (organizational and system); and among other outcomes showed a positive change in enhancing the policymakers’ research capacities for successful policymaking processes. However, according to Brownson and his colleague’s statements, when building capacity for EIPM, all levels should be included and there is no benefit in implementing interventions by “skipping one level of power” [43].

### 4.2. Research Engagement Actions

Over the years, in EIPM, a shift took place from opinion-based policies to action-based approaches where high-quality research evidence is used (accessed, appraised, and generated) [50]. These research engagement actions (REA) have been explored in the literature and it has been stated that improving policymakers’ research skills [62] and building personal relationships with researchers [58,63] are critical factors for REA and research use. The primary hypothesis of the SPIRIT Framework is that if “there is sufficient research capacity, and a reservoir of relevant and reliable research exists,” individuals and organizations may engage with research by accessing, appraising, or generating research, or by interacting with researchers. In this way, research use in policymaking will “come” as an inevitable outcome [7,48].

Crucial results from our survey in the domain of REA show that Danish policymakers are motivated and ready for change, but there are some obstacles impeding their research engagement. Although they reported skills and knowledge to access synthesized and primary research, as well as to appraise research, most of them (2/3) did not have a chance to be part of research projects conducted in their organizations, or in collaboration with researchers. Interestingly, the same number (2/3 of respondents) still plan for, support, and recommend undertaking future research. Here, we need more research on the organizational level to determine if the missing tools and systems in research use, collaboration, and EIPM are simply too invisible to the individual policymaker. Furthermore, more research is needed to determine if this accounts for the REA’s unsatisfactory evaluation.

As an additional factor influencing REA, the lowest score in our survey analysis was identified for “policymakers’ interaction with researchers,” where the data showed that the collaboration with researchers was rare for any of the critical activities, such as policy or research direction, development, or the implementation of joint research grants. Policymakers reported little opportunity to contribute to research publications or be part of a research team as advisors. However, most took part (as users) in dissemination activities, e.g., workshops, presentations, printed reports, or similar activities from and by researchers. The takeaway from previous literature is that bridging the gap between research and research-informed policy is “coming together at the same table”. Both parties (policymakers as consumers of research and researchers as research suppliers) should agree on what kind of knowledge is valid, needed, and in line with society’s health needs [34,62,64,65,66]. For example, the continuous, repeated communication and sometimes informal, personal relationships with researchers will raise mutual trust [67]. In addition, existing research shows that policymakers’ trust in research evidence is stronger towards researchers who are close to them and who sometimes work as internal organizational advisors, while there tends to be distrust towards those with whom they do not have regular close interaction [68]. International evidence suggests that collaborative research and continuous close partnerships and communication are essential facilitators of research dissemination and uptake [48,62,69].

### 4.3. Research Use

This study captures the extent to which research is used in different stages of policymaking. Study participants generally agreed that in each stage of health policymaking, research evidence was used on a limited scale. The previous national findings showed that research was used unsystematically where some of the barriers were: different policymakers’ research capacities, a lack of strong trust and relationships with researchers, and not enough locally relevant and directly usable research [57,58,60]. By studying these responses, which indicated that agenda-setting and policy development benefited most from the increased use of research, it is understandable why conceptual research use has been the preferred type of research used in policymaking.

Concerning the types of research used in different policy phases, the findings aligned partly with those reported by other surveys; that conceptual research use (to help understand and reflect on policy issues) was moderately to extensively used in health policymakers’ work [48,52,60]. In Danish research, conceptual modeling was mostly used in the policy cycle agenda, in the direction of demographic and statistical data to identify target groups, frame policies, and provide evidence-based guidelines and recommendations [56,60].

Furthermore, in line with the Australian survey [48], policymakers in our study showed an extensive instrumental (to decide about the content or direction of a health policy) and tactical use of research (to persuade others of a desired point of view or course of action). A 2013 Danish study, published by Jakobsen and colleagues, showed that single studies were used conceptually and instrumentally, while case studies and project reports were used to select policy development [60]. In this 2013 survey, however, the authors could not identify the types of research used in policy implementation and evaluation. A tactical type of research, not to inform decision making, but to justify policymaking direction, is often reported in the literature [54,70]. However, Makkar et al., in their article, concluded that tactical research use misuse research since it was selective and biased toward justifying needs and actions [71]. Makkar et al.’s advice was that organization leaders needed to have a clear position on research and the types of research use and communicate them to the policymakers. Moreover, their findings stated that conceptual and instrumental research informed the policy process, which preceded the policy decisions, while tactical research use came afterwards [72].

Imposed research use (the organization requires research use) was only rarely reported by the policymakers in our study. Additionally, many policymakers reported that the tools and systems to support research engagement actions and use either did not exist or that they were not aware that such tools existed.

### 4.4. Strengths and Limitations

This study had many strengths. To our knowledge, it reports the first extensive assessment on measuring the individual capacity to engage with and use research in Denmark and Europe in general. Second, to our knowledge, we were the first in Europe to use the newly developed SPIRIT Action Framework and the validated and reliable SEER measure [7]. This framework can help to understand and define the “weakest” areas of the health policy process when research use is in question. These results can be used for more needs-oriented, capacity-building strategies. Third, the survey was conducted with health policymakers who worked in organizations with different public health responsibilities and duties (e.g., municipality, region, NGOs, etc.), thereby increasing the generalizability of our findings. The findings showed which aspects of individual capacity catalyzed and influenced research use and which needed change.

The study also had limitations. Non-response to surveys is always an issue, especially when those who choose not to respond are different in some way, whether this is known or unknown, from the survey participants; the inferences from sample to population are therefore inappropriate. Given the present study’s recruitment approach via Danish public health organizations, which have other types of members beyond policymakers, and GDPR rules, which did not permit access to the membership lists (see the Methods Section), it was not possible to determine a response rate and evaluate the differences between participants and non-participants. Thus, even though the absolute number of policymakers who participated was among the largest in the context of European studies on a similar topic, we must assume some level of self-selection bias. Presumably, those who were more interested and motivated in the topic were more likely to take part, which might have led to an overestimation of the value that Danish policymakers assigned to the role of research in policymaking, as well as to their own skills to systematically integrate research.

## 5. Conclusions

The essential new knowledge about the health policy process among policymakers in Denmark clarifies the individual capabilities which need capacity-building interventions and the changes which are needed in the organizations to support individual decision makers in successfully translating research evidence into policy to improve population health outcomes. The study results could serve as a baseline for potential need-oriented interventions, even though it must be considered that the findings might not accurately reflect the status quo due to response bias.

The main conclusion from this study is that policymakers report having the capacity and motivation to use research in EIPM, but if and to what extent they are actually using these research capacities in their policymaking is questionable. They believe that their organizations need more research evidence in health policymaking, but they (the policymakers) are still unaware of the organizational tools and systems for research engagements and use, and the actual, concrete organizational research involvement in producing new evidence. The study results show that organizations need to develop a systematic communication about research with employees, and provide guidance for EIPM, support research use in policies, as well as define and develop guidelines for policy implementation and evaluations. Furthermore, organizational management needs to put in practice those tools and systems to systematically promote the interaction and research collaboration between researchers and policymakers, where context-oriented and ready-for-use research will be one of the outcomes. Moreover, it is essential to build networks and trusted partnerships between policymakers and researchers, which can be relied upon in the long term to support decision-making processes. Regarding the discrepancy between the reported research capacities and the actual research engagement actions, one could ask whether this is due to the organization not having a supportive environment for EIPM that permits the use of individual competencies, or whether the policymakers might have unclear concepts of what it means to practice and use EIPM. Furthermore, research that is extensively used by policymakers simply to justify a pre-decided policy direction raises a question about how public health policymakers should (or should not) find a balance between political loyalty and professional autonomy.

More in-depth qualitative research on these issues is needed, preferably under the SPIRIT Action Framework, using existing, validated qualitative tools such as: ORACLe (Organisational Research Access, Culture, and Leadership) and SAGE (Staff Assessment of Engagement with Evidence) [71,73]. Finally, we would like to stress that it is essential to properly define research capacity, research use in policymaking, as well as EIPM in general, and have a common definition to facilitate and improve understanding. If we do not act continuously, directly, and measurably and intervene on both individual and organizational levels, the evidence and use of research will experience great difficulty in finding a path into health policy.

## Figures and Tables

**Table 1:** Characteristics of study participants.

**Respondents’ characteristics**	***n* (%)**
Total:	91
**Gender:**	
Male	29 (32%)
Female	62 (68%)
**Age (years):**	
20–39	31 (34%)
40–49	37 (41%)
50+	23 (25%)
Highest level of education *:	
Short higher education	46 (51%)
Medium higher education	30 (33%)
Long higher education	15 (16%)
**Institutional affiliation**	
Municipality	40 (44%)
Regions	35 (38%)
other **	16 (18%)
**Working in present organization (in years)**	
0–5	48 (53%)
6 to 10	19 (21%)
11+	24 (26%)
**Years worked with health policy, program and/or strategy development**	
0–5	41 (45%)
6 +	50 (55%)
**Worked with evidence-informed policymaking in the last 12 months**	
Yes	56 (62%)
No	35 (38%)
**Ever worked as a researcher**	
Yes	47 (52%)
No	44 (48%)
**Working experience outside governmental organizations (in years)**	
less than 1 yr	44 (48%)
1 to 5	25 (28%)
6+	22 (24%)
Working experience in governmental organizations (in years) ***	
0–5	17 (19%)
6 to 10	24 (26%)
11+	50 (55%)

* Defined by Ministry of Education in Dk: https://ufm.dk/en/education/recognition-and-transparency/transparency-tools/europass/diploma-supplement/danish-higher-education-system-short-description. (accessed on 14 October 2021) ** Other/outside of governmental organization in the survey include non-governmental organizations, consultancy companies, private companies. *** Governmental organizations in the survey include municipalities, regions, and hospitals.

**Table 2:** Research Capacity, Research Engagement Actions and Research Use in Means, Standard Deviations (SD), Medians, Cronbach’ Alpha’s (*n* = number of responders).

	*n*	Mean	SD	Median	Cronbach’s Alpha
**Research Capacity**					
Subdomains:					
1. Value individual places on using research					
Sum score of seven items with continuous scales (1–5) divided by number of items	91 *	4.04	0.567	4.00	0.77
2. Confidence individuals have in his/her own knowledge and skills					
Sum score of seven items with continuous scales (1–5) divided by number of items	91 *	3.9	0.673	4.00	0.84
3. Value organization places on using research					
Sum score of five items with continuous scales (1–5) divided by number of items	91 *	3.41	1.142	3.6	0.91
4. Tools and systems that organization have to support research engagement actions and research use					
Sum score of seven items with continuous scales (1–4) divided by number of items	91 *	2.11	0.535	1.42	0.90
**Research Engagement Actions**					
Subdomain:					
1. Policymakers–researchers interaction in the last 12 months prior to survey					
Sum score of six items with continuous scales (1–4) divided by number of items	56 **	1.96	0.697	1.83	0.76
**Research Use in Policymaking**					
Subdomain:					
1. Extent of research use in policymaking in the last 12 months prior to survey					
Sum score of four items with continuous scales (1–6) divided by number of items	56 **	3.09	1.5	3.5	0.85

* Health policymaker members of the Danish Society of Public Health or public health coordinators in the Danish Healthy Cities Network. ** responders who had worked in writing, drafting, and developing health policies, strategies, or programs in the 12 months prior to survey.

**Table 3:** REA and RU in frequencies and percentages.

	Item/Question	Response	*n*	(%)
**Research Engagement Actions (REA)**				
Subdomains:				
1. Accessed synthesized research				
For area of policy or program:	Searched for reviews of research to summarize and evaluate the results of multiple studies (i.e., systematic reviews, meta-analyses)	No	22	(39%)
		Yes	34	(61%)
	Produced a review of research to summarize and evaluate the results of available studies	No	43	(77%)
		Yes	13	(23%)
2. Accessed primary research				
For area of policy or program:	Searched for research papers reporting the results of single studies (e.g., randomized controlled trials, qualitative studies)	No	24	(43%)
		Yes	32	(57%)
	Searched for research on government websites	No	10	(18%)
		Yes	46	(82%)
3. Appraised research *				
For policy or program work, assessment of the usefulness of the research or review was based on:	The appropriateness of methods used to answer the question	No	17	(35%)
		Yes	31	(65%)
	The likelihood that the methods used meant that the results were reliable (unbiased)	No	14	(29%)
		Yes	34	(71%)
	The generalizability of the findings to your context, based on similarity of the included population, health system or other factors	No	3	(6%)
		Yes	45	(94%)
4. Generated research				
For policy or program work did you:	Undertake or participate in an internally conducted research project or analysis of data	No	38	(68%)
		Yes	18	(32%)
	Commission or partner with researchers to conduct a research project or analysis of data	No	39	(70%)
		Yes	17	(30%)
	Plan or undertake an evaluation of the program or policy	No	29	(52%)
		Yes	27	(48%)
	Advocate for research to be undertaken in the future	No	19	(34%)
		Yes	37	(66%)
**Research Use in Policymaking (RU)**				
Subdomain:				
Type of research use				
Type of research used for the area of policy or program that policymaker was involved in during the last 12 months prior to survey	Conceptual research use (to help understand how to think (reflect) about an issue)	No	32	(57%)
		Yes	24	(43%)
	Instrumental research use (to decide about content or direction of a policy or program)	No	8	(14%)
		Yes	48	(86%)
	Tactical research use (to persuade others to a point of view or course of action)	No	10	(18%)
		Yes	46	(82%)
	Imposed research use (because your organization required you to use research)	No	41	(73%)
		Yes	15	(27%)

* answers by respondents who had reported they found relevant research for development of health policy. Eight of them did not find relevant research, so they were excluded from analysis for this question.

## Data Availability

The data presented in this study are available on request from the corresponding author. The data are not publicly available due to an ongoing results analysis for a new article.

## Supplementary Materials

The following are available online at https://www.mdpi.com/article/10.3390/ijerph182111014/s1, File S1: Annex I Descriptions of Variables, File S2: Annex II Summed up continuous variables from three principal domains, File S3: Annex III Collapsed number of categories of demographic variables, File S4: Annex IV Item Descriptive Statistics for SEER Capacity Scales (Predisposing Factors).
